# Better Together: Tools to Aid Patient and Public Involvement in Evidence Synthesis and the Need for Consensus on Essential Reporting Items

**DOI:** 10.1002/cesm.70087

**Published:** 2026-06-01

**Authors:** Eugenie Evelynne Johnson, Fiona Pearson, Danielle Pollock, Jack S. Nunn, Richard Morley, Sophie Staniszewska, Andrea C. Tricco, Alex Todhunter‐Brown

**Affiliations:** ^1^ Population Health Sciences Institute Newcastle University Newcastle upon Tyne UK; ^2^ PenTAG University of Exeter Exeter UK; ^3^ Honorary Principal Research Associate, Population Health Sciences Institute Newcastle University Newcastle upon Tyne UK; ^4^ HESRI (Health Evidence Synthesis Recommendations and Impact) University of Adelaide Adelaide Australia; ^5^ Science for All Melbourne Australia; ^6^ Public Contributor UK; ^7^ Warwick Applied Health, Warwick Medical School University of Warwick Coventry UK; ^8^ Epidemiology Division and Institute of Health Policy, Management and Evaluation University of Toronto Dalla Lana School of Public Health Ontario Toronto Canada; ^9^ Li Ka Shing Knowledge Institute of St Michael's Hospital Unity Health Toronto Ontario Toronto Canada; ^10^ Glasgow Caledonian University Glasgow UK

**Abbreviations**
ACTIVE: Authors and Consumers Together Impacting on eVidencE
CHEERS: Consolidated Health Economic Evaluation Reporting Standards
CIHR: Canadian Institutes of Health Research
CONOSRT: Consolidated Standards of Reporting Trials
EQUATOR: Enhancing the QUAlity and Transparency Of health Research
GRIPP2: Guidance for Reporting Involvement of Patients and the Public 2
MuSE: Multi‐IntereSt‐holder in Engagement consortium
MuSE‐ES: Multi‐IntereSt‐holder in Engagement consortium Evidence Synthesis project
NIHR: National Institute for Health and Care Research
PEIRS: Patient Engagement In Research Scale
PiiAF: Public Involvement Impact Assessment Framework
PIRIT: Public Involvement in Research Impact Toolkit
PPI: patient and public involvement
RIPPLES: Rapid Involvement of Patients and the PubLic in Evidence Synthesis
SPIRIT: Standard Protocol Items: Recommendations for Interventional Trials
STARDIT: Standardised Data on Initiatives
UNESCO: United Nations Educational, Scientific and Cultural Organization

## 1 Introduction

Globally, there is a growing imperative for patients and the public to be involved in research. The United Nations Educational, Scientific and Cultural Organization's (UNESCO) 2021 Recommendation on Open Science called for opening the “processes of scientific knowledge creation, evaluation and communication” beyond the academic community, which many countries are now acting upon [1]. In the UK, the National Institute of Health and Care Research (NIHR) expects that all research involves patients and the public, recently publishing its Strategic Commitments for Public Partnerships 2025–2030 [2]. In Canada, the Strategy for Patient‐Oriented Research (SPOR) aims to transform patients into proactive partners within research [3], while in the US the Patient‐Centred Outcomes Research Institute (PCORI) have developed six foundational principles for partnerships within research, including with patients and communities [4]. There has also been an increasing visibility of patient and public involvement (PPI) within health research within Nordic countries [5], as well as Australia [6], and the MuSE Consortium is an international group of over 100 individuals interested in engagement in research and guidelines [7]. Furthermore, the recent GIN‐McMaster Guideline Development Checklist Extension for Engagement furthers the international imperative for involvement, including PPI, by providing a transparent reporting guideline for the inclusion of interest‐holders in guideline development [8].

Terminology in this field varies, but there have been recent attempts to reach consensus on shared terms and definitions [9]. Building on these initiatives, within this paper we use the term “interest‐holders” to mean people with a personal, professional or financial interest in research, including those who may be directly or indirectly affected by the research [9]. We use the abbreviation “PPI” to mean the involvement of interest‐holders who identify as patients, family or informal caregivers of patients, or members of the public. The NIHR defines PPI as research that is carried out “with” or “by” people, rather than “about” them [10].

Evidence syntheses, research which brings evidence together using robust methodologies, are central to informing decisions of key interest‐holders. There is a range of different types of evidence syntheses, including quantitative and qualitative systematic reviews, scoping reviews, rapid reviews and evidence gap maps [11]. Despite recognized variations in terminology, the importance of PPI within evidence syntheses has been strongly supported for across international organizations. Notably, Cochrane, often cited as the “gold standard” for systematic reviews, were early adopters of PPI and have a long‐standing history of involvement in their reviews [12]. Cochrane later funded the Authors and Consumers Together Impacting on eVidencE (ACTIVE) project in 2016, launching the Cochrane Co‐Production Methods Group in 2024 with the aim of driving forward methodological developments in the area, and maintaining its Patient and Public Network [13, 14]. The JBI have also published guidance on involving knowledge users within scoping reviews to move from only consulting with interest‐holders to co‐creation processes [15]. Furthermore, there are clear benefits to involvement for evidence syntheses. Patients and the public have reported being able to contribute meaningfully to evidence syntheses by shaping outcomes and future projects as well as feeling empowered by the process, while researchers have stated that PPI enhances the overall quality and relevance of evidence syntheses [16].

Despite this, there remains considerable scope and challenge to embed PPI more consistently. Guidance to conduct meaningful PPI is crucial, particularly given the noted benefits for evidence syntheses, researchers and patients and members of the public. A recent project to identify the top research priorities relating to the methods of involving patients and public in co‐producing evidence syntheses found that several priorities related to the need for clear tools and guidance for PPI in evidence synthesis [17].

Here, we aim to introduce and discuss current tools suitable for supporting PPI in evidence synthesis. Our goal is to facilitate understanding of key tools, their similarities and differences, encourage use of the same tools by those producing evidence syntheses and highlight any major gaps within our methodological understanding.

## 2 Current Tools for Embedding PPI Into Evidence Synthesis

There are currently some key conceptual frameworks researchers can use when considering how to conduct PPI within evidence syntheses. Table 1 summarizes four key tools that we, individuals with a range of backgrounds involved in evidence syntheses, and members of key organizations such as Cochrane Co‐production Methods Group, have helped develop and believe have the potential to be core tools for supporting PPI in evidence synthesis. However, these may not be exhaustive.

**Table 1:** Key tools for embedding PPI into evidence syntheses.

Tool	Description
**Evidence‐synthesis specific**	
Authors and Consumers Together Impacting on eVidencE (ACTIVE) framework	The ACTIVE Framework was developed to provide a structure to describe key components of interest‐holder involvement within evidence syntheses. The framework comprises five key domains: who was involved; how interest‐holders were recruited; the mode of involvement; the stages of the review process in which they were involved; and the level of involvement at each stage [18].
Rapid Involvement of Patients and the PubLic in Evidence Synthesis (RIPPLES) framework	This was developed in response to the growing emphasis on more rapid evidence reviews, particularly since the COVID‐19 pandemic, with a focus on supporting meaningful PPI when timelines and resources are constrained. Intended as a companion to ACTIVE, RIPPLES provides guidance on building the foundations for impactful PPI as well as embedding the perspectives of patients and the public into rapid projects [19]. Further elaboration is in preparation, designed to provide more in‐depth context to support future use of this framework.
**Not evidence synthesis‐specific**	
Guidance for Reporting Involvement of Patients and the Public 2 (GRIPP2)	While not specifically focussed on evidence syntheses, GRIPP2 provides an important framework designed to support the reporting of the impact of PPI on health research [20]. Used alongside ACTIVE and/or RIPPLES, GRIPP2 can support clearer reporting of the aim, methods and impact of the PPI, whilst ACTIVE provide a framework to clearly describe details of who was involved, the activities they contributed to, and the stages of the evidence synthesis at which there was involvement.
Standardised Data on Initiatives (STARDIT)	A platform aiming to address current limitations and inconsistencies in sharing data. It provides a standardized reporting system across initiatives and disciplines, including who was involved, what tasks they did and any observed impacts [21]. STARDIT has been proposed as a multi‐lingual tool for collaboratively reporting data about PPI in evidence synthesis [22]. Uniquely, STARDIT can be used to report data from multiple different reporting standards, including PRISMA standards.

Abbreviations: ACTIVE, Authors and Consumers Together Impacting on eVidencE; GRIPP2, Guidance for Reporting Involvement of Patients and the Public 2; PPI, patient and public involvement; PRISMA, Preferred Reporting Items for Systematic reviews and Meta‐Analyses; RIPPLES, Rapid Involvement of Patients and the PubLic in Evidence Synthesis; STARDIT, Standardised Data on Initiatives.

Beyond these, a larger body of guidance exists to be able to aid review authors in embedding meaningful PPI into their work. From the UK, the NIHR provide numerous guidance notes relating to best practice for PPI, including briefing notes for researchers and INCLUDE, which aid researchers looking to conduct meaningful PPI across a broad range of health and social care methodologies [10, 23]. Resources also exist regarding interest‐holder involvement beyond health and social care [24]. As it is equally important to document and report the impact of PPI within research, freely‐available methods of reporting the impact of PPI on research include the Public Involvement Impact Assessment Framework (PiiAF), the Public Involvement in Research Impact Toolkit (PIRIT) and the Patient Engagement In Research Scale (PEIRS) [25, 26, 27]. Our team is also aware of some other important new developments. This includes MuSE‐ES, a large project funded by the Canadian Institutes of Health Research (CIHR) to bring together up‐to‐date evidence on all aspects of interest‐holder involvement within evidence syntheses. A protocol to develop equity‐oriented guidance to conduct, evaluate and report engagement in evidence syntheses was published in 2023 [28].

## 3 Mind the Reporting Gap: The Case for Improved Reporting of PPI

The aforementioned tools and frameworks highlight just some of the guidance available to reviewers looking to embed PPI into evidence syntheses. However, despite the increase in available tools and guidance for review authors, there remains a substantial lack of transparent reporting of meaningful and inclusive PPI methods within evidence syntheses [29]. An analysis of papers published in a single journal between 2015 and 2022 found that 25.8% of 217 systematic reviews reported including PPI [30]. This challenge only becomes more pronounced within rapid evidence syntheses; another cross‐sectional study reported that only 5.8% of 103 sampled rapid reviews involved patient partners in their processes [31]. Furthermore, a recent scoping review concluded that only 19% of 272 evidence syntheses which reported interest‐holder involvement provided a comprehensive description of the method or approach to involvement. Further, this study found that 10% of 272 evidence syntheses did not report any information about who was involved and 25% of 272 were unclear as to when in the review process interest‐holders had been involved [32]. These examples highlight key gaps in reporting of PPI within evidence syntheses.

Given the importance of evidence syntheses in policy and decision‐making processes, the need for transparent reporting of PPI within the methodology cannot be understated. Although tools such as GRIPP2 provide a template for how PPI can be reported across health and care research [20], we currently do not have a robust tool for reporting PPI across different types of evidence syntheses spanning multiple disciplines. The Preferred Reporting Items for Systematic reviews and Meta‐Analyses (PRISMA) checklists are well‐established and routinely used to report key methods used within a range of evidence syntheses. However, in contrast to other methodologies, PRISMA currently does not have an extension or items relating to PPI. The 2025 Consolidated Standards of Reporting Trials (CONSORT) and Standard Protocol Items: Recommendations for Interventional Trials (SPIRIT) guidance for randomized controlled trials both contain items relating to PPI [33, 34], while the Consolidated Health Economic Evaluation Reporting Standards (CHEERS) checklist for reporting of economic evaluations was also recently updated within an item regarding PPI [35].

As such, we believe there is a need to develop a robust reporting guideline for all evidence syntheses to transparently detail PPI activities and their impact. Developed using robust and established methodologies, such as those from the EQUATOR Network [36], such a guideline could potentially include domains such as reporting who was involved, when, the methods used to facilitate involvement, and the impact of involvement. This reporting guideline would: standardize how PPI is reported across evidence syntheses; promote overall transparency in methods used; increase accountability to interest‐holders; and encourage best practice in PPI methods.

While we strongly advocate for the development of a robust, consensus‐driven reporting guideline, we also acknowledge that this will take time. During this period, we encourage journal editors, funders and review authors to not only meaningfully involve patients and the public within evidence syntheses but also more transparently report the quality and consistency of involvement within manuscripts [29]. It may also mean journals themselves encouraging standalone sections within manuscripts to document PPI; Cochrane have already added a reporting rubric for PPI to their reviews as standard. In doing so, we can continue to make inroads into the transparent reporting of PPI within evidence syntheses while venturing towards agreed items for preferred reporting of PPI in evidence syntheses.

## 4 Conclusions

Despite the known mutual benefits of PPI for both review authors and those involved, and a range of tools and resources to help embed PPI into reviews, there are still substantial challenges regarding the reporting of PPI within evidence syntheses. It is therefore time to make significant improvements in how PPI is reported within evidence syntheses across disciplines through the development of a robust and co‐created reporting standard.

## Author Contributions

**Eugenie Evelynne Johnson:** conceptualization, writing – original draft. **Fiona Pearson:** conceptualization, writing – review and editing. **Danielle Pollock:** writing – review and editing. **Jack S. Nunn:** writing – review and editing. **Richard Morley:** writing – review and editing. **Sophie Staniszewska:** writing – review and editing. **Andrea C. Tricco:** writing – review and editing. **Alex Todhunter‐Brown:** conceptualization, writing – review and editing.

## Funding

The authors have nothing to report.

## Conflicts of Interest

EEJ conceptualized and co‐developed the RIPPLES framework for patient and public involvement within rapid evidence syntheses, which was funded by the National Institute of Health and Care Research (NIHR) Innovation Observatory. Both EEJ and FP have been part of the National Institute for Health and Care Excellence (NICE) Technology Appraisal Group at Newcastle University, funded by the NIHR. FP also assisted in co‐developed the RIPPLES framework, funded by the NIHR Innovation Observatory. FP is now an employee of Adelphi Values PROVE™ (Bollington, England, UK), is a member of the INAHTA Qualitative Methods Special Interest Group and has been part of the Evidence Assessment Group (EAG) funded by NICE. DP has received support from Cocrhane for flights and accommodation to attend the Cochrane Leadership Meeting in Lisbon (2025) and has received support for flights, accommodation and registration costs to attend workshops and the waves of change conference in 2024 and 2025 from CRE Stillbirth. JSN had their travel paid by Cochrane to attend the Global Evidence Sumit in Prague, Czech Republic, in 2024. RM was a salaried employee as the Consumer Engagement Officer for Cochrane from August 2015 until July 2024. SS has various contracts with the NIHR and the Medical Research Council in the UK and has had roles with Health Data Research UK and NICE. ACT is funded by a Tier 1 Canada Research Chair in Knowledge Synthesis for Knowledge Users. ATB has received NIHR grant funding as co‐lead of the NIHR Evidence Synthesis Scotland InitiativE (NESSIE Evidence Synthesis Group) and Canadian Institutes of Health Research (CIHR) as co‐investigator of the MuSE‐ES project. ATB is also an unpaid co‐lead of the Cochrane Co‐Production Methods Group and led the development of ACTIVE framework.

## Data Availability

Data sharing not applicable to this article as no datasets were generated or analyzed during the current study.
